# Supplementary material for: Generation and annihilation time of magnetic droplet solitons
Source: Sci Rep. 2018 May 1;8:6847. doi: 10.1038/s41598-018-25134-z (PMC5931510; doi:10.1038/s41598-018-25134-z)
Supplement: Supplementary file 1 — Supplementary Section [file 41598_2018_25134_MOESM1_ESM.pdf]

Supplementary Section:

Generation and annihilation time of magnetic droplet solitons

Jinting Hang,[1] Christian Hahn,[1] Nahuel Statuto,[2,3] Ferran Macià,[2,3] and Andrew D. Kent[1]

[1]Center for Quantum Phenomena, Department of Physics,

New York University, New York, New York 10003 USA

[2]Department of Condensed Matter Physics,

University of Barcelona, 08028 Barcelona, Spain

[3]Institut de Cincia de Materials de Barcelona (ICMAB-CSIC),

Campus UAB, 08193 Bellaterra, Spain

I. HYSTERESIS

In Fig. 1 we show a state diagram of a 100 nm diameter nanocontact — the same nanocontact used in the experiments described in the main text — determined from slow (quasistatic) resistance versus current measurements as a function of applied perpendicular magnetic field. A step increase/decrease in resistance is associated with the creation/annihilation of the droplet state, as shown in Fig. 1(b) of the main article; the current at which the resistance step occurs was used to create the state diagram, indicating regions in which the droplet mode can be excited. In the bistable zone, hysteretic behaviour is observed. A dashed line indicates the field at which the pulsed current experiments were carried out.

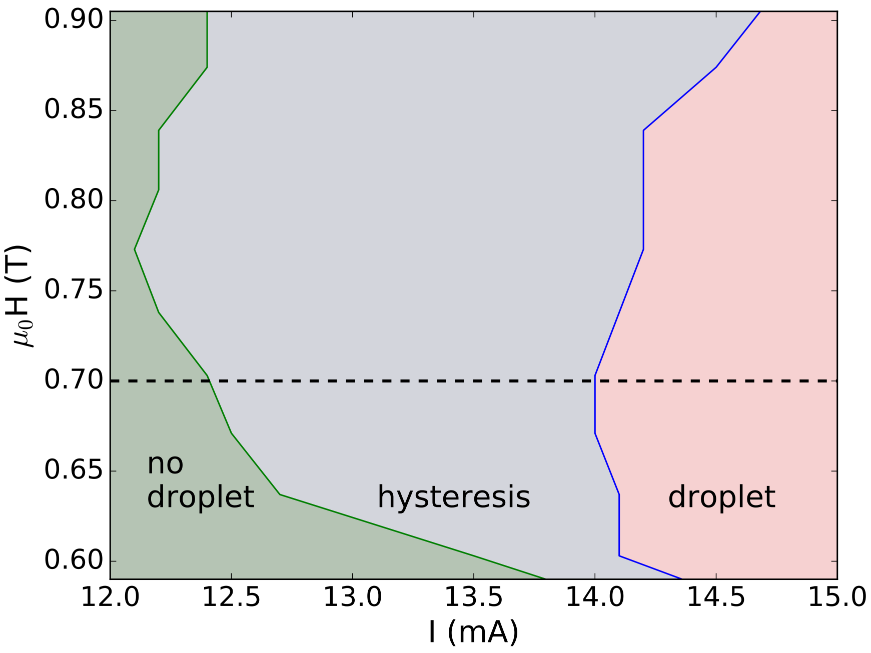

FIG. 1: **Nanocontact state diagram.** State diagram of the 100 nm diameter nanocontact sample used in the experiments reported in the main text as a function of current and out-of-plane field, indicating regions where the droplet mode is excited. The bistable zone where both droplet and no droplet states are allowed and hysteretic behaviour is observed are also shown.

II. SPECTRAL MEASUREMENTS

Fig. 2 shows the dc and high-frequency voltage response measured in a nanocontact with diameter of 100 nm, again, the same sample as used in the experiments reported in the main text. These characteristics, as shown in previous studies of droplet solitons, are indicative of the dynamic nature of the soliton modes. We see at the onset current, $I_c \simeq 14$ mA, there is a step increase in dc resistance and a corresponding abrupt decrease of the signal frequency to $f \simeq 20$ GHz at an applied field of $\mu_0 H = 589$ mT. The step in resistance and the decrease of the signal frequency is associated with the creation of a droplet soliton. We also show in Fig. 2 a sweep down of the applied current showing the existence of hysteresis. We note that the dc and spectral measurements were done in different current sweeps.

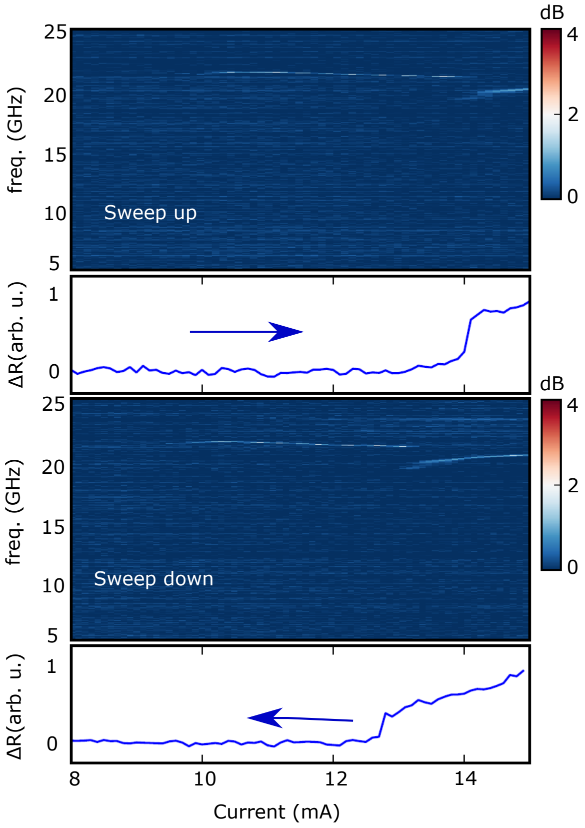

FIG. 2: **Spectral measurements of droplet states** (Top panels) High-frequency spectra as a function of applied current at an applied field of 589 mT. (Bottom panels) DC resistance as a function of current for the same sample. The two measurements (spectral and dc resistance) were taken on a 100 nm diameter nanocontact in separate current sweeps.

III. DROPLET GENERATION AND ANNIHILATION DATA ON A LARGER DIAMETER NANOCONTACT

Data taken on an another sample with a 150 nm diameter nanocontact is shown in Fig. 3. Fig. 3a shows the bistable zone in a current sweep at a fixed perpendicular applied field of 0.7 T. Droplet generation occurs in the up-sweep (red curve) and annihilation occurs in the down-sweep (blue curve). Fig. 3b shows that up to $\sim$ 10 μs pulses are needed to generate droplet solitons. Due to the rf characteristics of the bias-T used, pulse durations beyond 20 μs are distorted, causing a decrease in pulse amplitude with time and a negative going portion after the pulse. The distortion of longer pulses causes the reduction of the generation probability for long pulses.

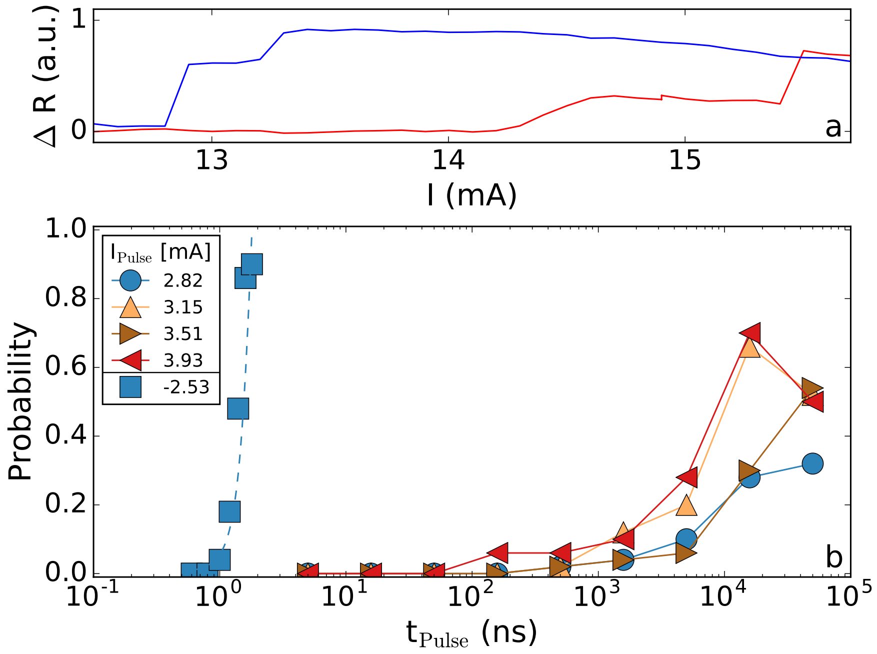

FIG. 3: **Droplet annihilation and generation results. a,** Resistance versus current hysteresis loop in an applied perpendicular field of 0.7 T. **b,** Annihilation (blue squares) and generation probabilities on the same sample.

IV. DROPLET DEPHASING

Our droplet simulations show an instability that leads to displacement of the droplet from the nanocontact region, similar to those reported in Ref.[1]. Once displaced, the droplet rotates around the nanocontact region with a frequency in the kHz range (see video 1). Figure 4 shows the time evolution of the magnetization inside the nanocontact region during the dephasing process. The images in this figure show the spins at the droplet boundary before and after dephasing.

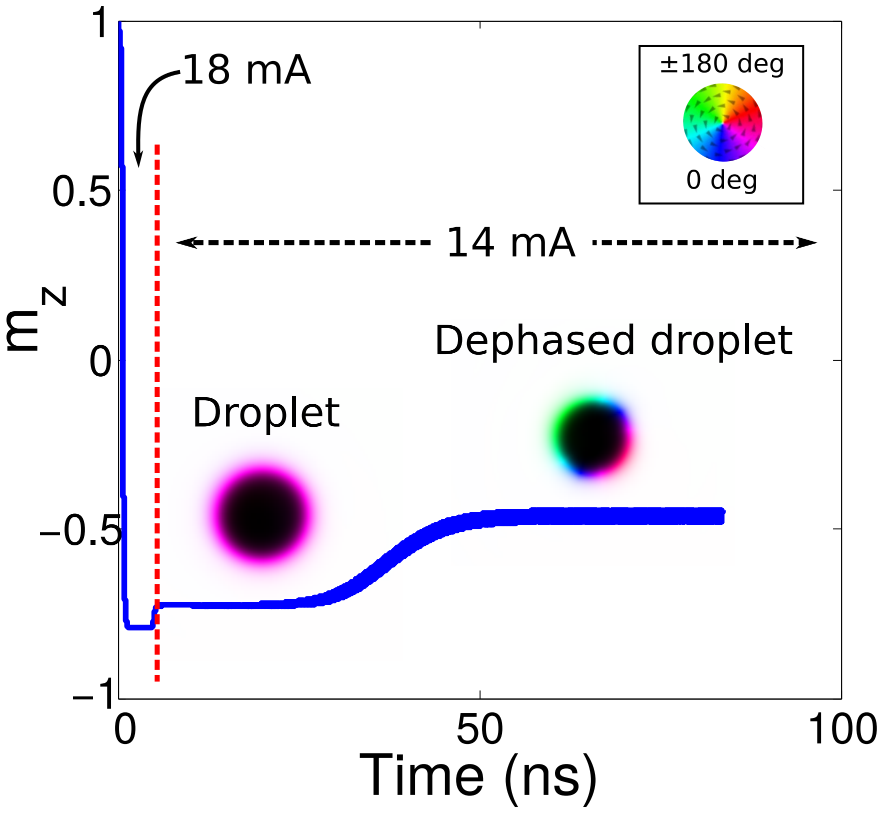

FIG. 4: **Time evolution of the magnetization inside the nanocontact**. A droplet is created with a current of 18 mA and after 10 ns the current is changed to 13.8 mA (and the corresponding Oersted fields changed as well). The droplet takes a few nanoseconds to reach a new steady state. After an additional 20 to 25 ns, the droplet begins to drift out of the nanocontact and because of an asymmetry caused by the Oersted fields, it dephases. The color scale indicates the direction of the spins at the droplet boundary and show the droplet with the spins at its boundary in phase and dephased.

V. WAITING TIME IN DROPLET GENERATION

To simulate droplet generation the magnetization is initialized with a small angle to the film normal and allowed to relax towards equilibrium for different times before applying the current pulse. Figure 5 shows the waiting time, a measure of the time needed for the creation and annihilation process, with different relaxation times as a function of the current pulse amplitude. We define the waiting time t_W as the time between the application of the pulse and the time with maximum variation of magnetization, calculated as the maximum of the derivative of the magnetization curve, m_z, of the nanocontact versus time. We simulate droplet generation with the same pulses conditions but with different initial magnetization relaxation times 1.5, 3 and 10 ns. A longer relaxation time leads to a smaller initial displacement of the magnetization from the equilibrium direction and thus a smaller initial spin-transfer torque. The result shows how the waiting time depends strongly on the initial magnetization state.

The time it takes to annihilate a droplet—defined as the time from the onset of the pulse to that of the maximum variation of magnetization—shows a very different behavior. This time also does not depend on the relaxation time. Figure 5 shows a clear asymmetry between droplet generation and annihilation processes. The waiting time for the creation process is reduced if the initial magnetization state is strongly tilted, as indicated by the dashed grey line in Fig. 5. This data was obtained by the same procedure as that used to obtain the blue curve, with 1.5 ns of relaxation, but with a small in-plane field (5 mT) applied during the creation process, to tilt the magnetization away from the z axis. The inset of Fig. 5 shows an overlay of the time evolution of magnetization for generation events of different pulse amplitudes, shifted by their individual waiting time. This shows that the actual generation process is fast and self-similar: the main contribution to the droplet generation time is the time it takes to build up an initial magnetization angle.

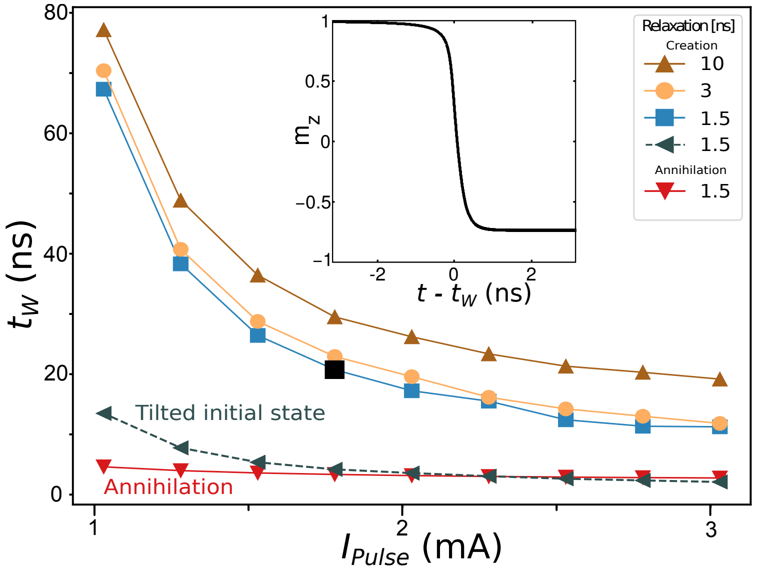

FIG. 5: **Waiting time for annihilation and creation process as a function of the pulse amplitude.** The red curve shows the annihilation time for different current pulse amplitudes. In time to annihilate the droplet does not dependent strongly on the current pulse amplitude. The other curves represent the creation processes. The waiting time increases exponentially when the pulse amplitude is reduced and also shows a dependence on the initial state of the magnetization. Different curves are for different initial magnetization states, achieved by using different relaxation times before applying the pulse; pulses were applied after 1.5, 3 and 10 ns of magnetization evolution. The dashed grey line represent a creation process using the same procedure as the blue curve with 1.5 ns of relaxation but with a small in-plane field (5 mT) applied during the current pulse. The inset represents all creation curves, which collapse to one curve when are shifted by their correspondent waiting time. The black square represents the curve in Fig. 4b, right panel, in the main text.

VI. SPEEDING UP THE CREATION PROCESS OF DROPLETS

An interest in applications is the possibility of operating at high speeds and, thus, a waiting time of several hundreds of nanoseconds for droplet creation is a concern. Here we show that *i)* that the creation process without the waiting time is indeed as fast as the annihilation process taking about 1 ns and *ii)* the waiting time can be reduced and eliminated.

Figure 6 shows three different curves for droplet annihilation corresponding to points from Fig. 5. In Fig. 6 we also plot a creation curve (recall that all creation curves collapse to a single curve when the waiting time is subtracted, as shown in the inset of Fig. 5) in order to show that the time it takes the magnetization to reverse is indeed similar between creation and annihilation.

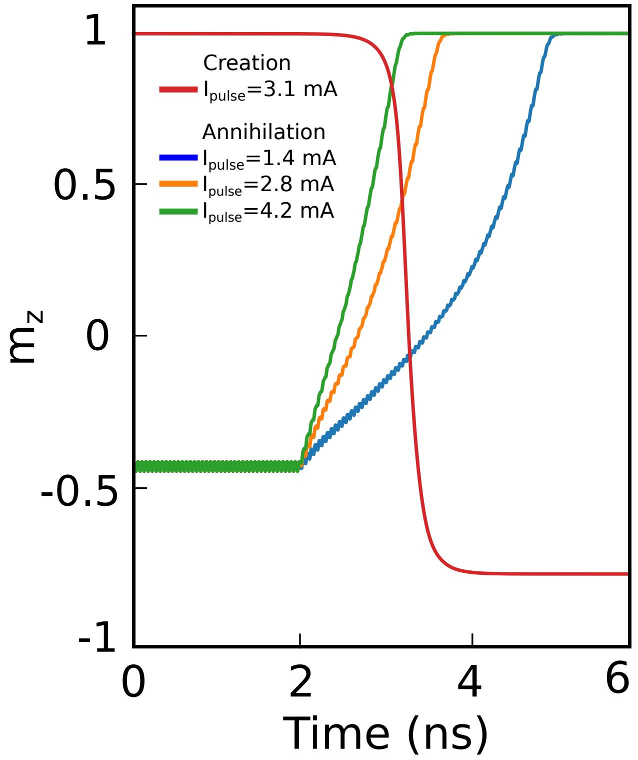

FIG. 6: **Creation and annihilation curves**. Time-evolution curves representing the annihilation, red, green and blue, and the creation, black, of a droplet. After 2 ns the current is changed to lower currents for the annihilation and to higher currents for the creation. Different annihilation curves have different pulse amplitudes. The black curve represents creation curves, which are equal for different amplitudes of the pulse when shifted by their correspondent waiting time (see inset of Fig. 5).

Next we show that the initial value of applied current is less relevant than the magnetization state. In the main manuscript we focused on a initial magnetization state with an applied current of 13.8 mA that corresponded to a bistable state. The reason was that the experiment aimed to compare both creation and annihilation times departing from a same initial state in applied current. However, it is clear that for any application there is no need to maintain an applied current to sustain a non-droplet state. Figure 7a shows a comparison of the evolution curves for a droplet creation with different initial states: a bistable state with applied current of 13.8 mA and a state with no applied current. We see that the waiting time for the no-applied current initial state is larger than for the bistable state with applied current. The reason is that the initial magnetization state is not the same.

Finally, we show that by applying a short pulse of magnetic field that destabilizes the initial state we eliminate the waiting time. Figure 7b shows the same comparison from Fig.

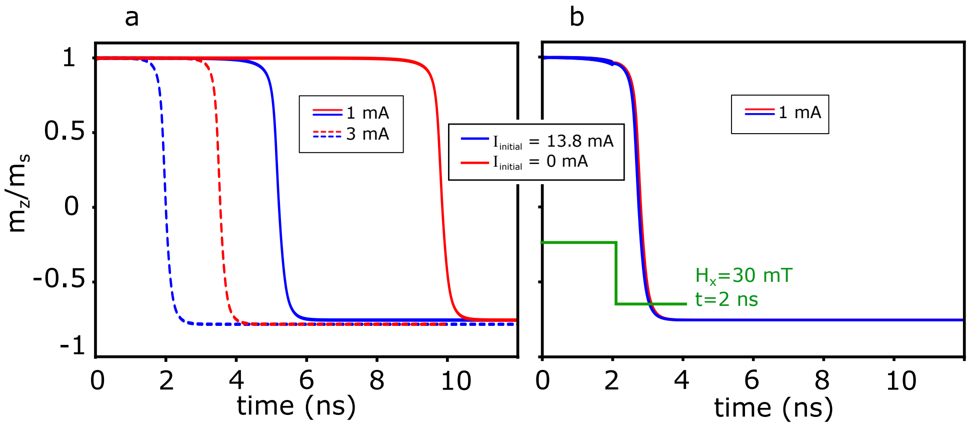

FIG. 7: **Creation curves as function of initial state.** a) Shows a comparison of the evolution curves for a droplet creation with different initial states: in blue for an initial bistable state corresponding to $I = 23.8$ mA and in red for an initial stat with applied current. Two cases are compared with different pulse amplitudes (1 (14.8) mA in straight lines and 3 (16.8) mA in dashed lines. b) Shows the same comparison as a) for the pulse of 3 (16.8) mA and with an initial field pulse of 2 ns and 30 mT).

7a with 3 mA and with an initial 30 mT field pulse with a duration of 2 ns. We see that both curves reduced the waiting time from 6 and 10 ns to about 2 ns and both curves basically match. This is because the field pulse tilts the magnetization and makes the spin torque more effective from the moment the current pulse is turned on.

VII. MICROMAGNETICS CODE

```
//Mumax Code

// mumax3 is a GPU-accelerated micromagnetic simulation open-source software
// developed at the DyNaMat group of Prof. Van Waeyenberge at Ghent University.
// The mumax3 code is written and maintained by Arne Vansteenkiste.

//GRID
LL := 256
Lz := 1
SetGridSize(LL, LL, Lz)
SetCellSize(3e-9, 3e-9, 4e-9)
SETPBC(4, 4, 0)

//NANOCONTACT
diam_circ := 100e-9
r_circ := diam_circ / 2
A_circ := pi * pow(r_circ, 2)

DefRegion(1, layer(0).intersect(circle(diam_circ)))
Tableadd(m.region(1))

//MATERIAL PROPERTIES
 lambda = 1              //Slonczewski parameter
 epsilonprime = 0        // Slonczewski secondairy STT term
 msat = 500e3            //Saturation
 ku1 = 200e3             //Uniaxial Anisotropy
 anisU = vector(0, 0, 1)
 Aex = 10e-12            //Exchange
 alpha = 0.03            //Damping

    //OERSTED FIELDS———————
posX := 0.
posY := 0.
mask := newSlice(3, LL, LL, Lz)
current := vector(0., 0., 1.)

for i := 0; i < LL; i++ {
        for j := 0; j < LL; j++ {
            r := index2coord(i, j, 0)
            r = r.sub(vector(posX, posY, 0))
            b := vector(0, 0, 0)
            if r.len() >= r_circ {
              b = r.cross(current).mul(mu0 / (2 * pi * r.len() * r.len()))
            }
        else {
              b = r.cross(current).mul(mu0 / (2 * pi * r_circ * r_circ))
            }
        for k := 0; k < Lz; k++ {
            mask.set(0, i, j, k, b.X())
            mask.set(1, i, j, k, b.Y())
            mask.set(2, i, j, k, b.Z())
            }
        }
}
```

```
//END OERSTED———————

//INITIAL CONDITIONS
angle := 85.
my := cos(angle * pi / 180)
mz := sin(angle * pi / 180)
m = Uniform(0, my, mz)

Value_Bext_z := 1.1 //external field in z direction
B_ext = vector(0., 0, Value_Bext_z) //external field
fixedlayer = vector(0.,0.,1.)
Curr := -13.8e-3          //current in Amps
Pol = 0.21
TableAddVar(Curr, "Current", "A")

//SAVING
Tableautosave(5e-13)
Autosave(m,1e-9)

//RUNNING
 Relax()
 Curr = -13.8e-3
 j.SetRegion(1, vector(0, 0, Curr/A_circ*Lz)) //current
 B_ext.RemoveExtraTerms()
 B_ext.add(mask, Curr)                        // Oersted fields
 run(1.5e-9)                                  //running 3 ns

 Curr = -13.8e-3 - 1.73e-3                    //pulse of +1.73 mA
 j.SetRegion(1, vector(0, 0, Curr/A_circ*Lz))
 B_ext.RemoveExtraTerms()
 B_ext.add(mask, Curr)
 run(21.0e-9)                                 //running 21 ns

 Curr = -13.8e-3
 j.SetRegion(1, vector(0, 0, Curr/A_circ*Lz))
 B_ext.RemoveExtraTerms()
 B_ext.add(mask, Curr)
 run(100e-9)                                  //running 100 ns

 Curr = -13.8e-3 + 1.73e-3                    //pulse of -1.73 mA
 j.SetRegion(1, vector(0, 0, Curr/A_circ*Lz))
 B_ext.RemoveExtraTerms()
 B_ext.add(mask, Curr)
 run(1.3e-9)                                  //running 1.3 ns

 Curr = -13.8e-3
 j.SetRegion(1, vector(0, 0, Curr/A_circ*Lz))
 B_ext.RemoveExtraTerms()
 B_ext.add(mask, Curr)
 run(10e-9)                                   //running 10 ns
```

---

[1] S. M. Mohseni, S. R. Sani, J. Persson, T. N. A. Nguyen, S. Chung, Y. Pogoryelov, P. K. Muduli, E. Iacocca, A. Eklund, R. K. Dumas, et al., Science **339**, 1295 (2013)
